# Effect of repeated bouts versus a single bout of moderate‐intensity exercise on postexercise inhibitory control

**DOI:** 10.14814/phy2.14528

**Published:** 2020-08-09

**Authors:** Takeshi Sugimoto, Tadashi Suga, Hayato Tsukamoto, Keigo Tomoo, Kento Dora, Takeshi Hashimoto, Tadao Isaka

**Affiliations:** ^1^ Faculty of Sport and Health Science Ritsumeikan University Kusatsu Shiga Japan

**Keywords:** brain health, cognitive function, exercise adherence, lactate

## Abstract

We previously demonstrated that duration of aerobic exercise plays an important role in improving cognitive inhibitory control (IC). Repeated bouts of aerobic exercise (R‐EX), which are performed with a rest interval, is a useful strategy in improving physical health parameters in similar manners to a single bout of aerobic exercise (S‐EX). However, whether R‐EX would be effective in improving IC remains unknown. This study compared the effect of R‐EX versus S‐EX of moderate‐intensity exercise on postexercise IC. Twenty healthy, young males performed both R‐EX and S‐EX in a crossover design. R‐EX consisted of two 20‐min moderate‐intensity bouts (60% of peak oxygen consumption) for 20 min_,_ which were separated by a 20‐min rest interval. S‐EX consisted of a once‐off 40‐min moderate‐intensity bout without rest interval. To evaluate IC, the color‐word Stroop task was administered before exercise, immediately after exercise, and every 10 min during the 30‐min postexercise recovery period. The reverse‐Stroop interference score, which is a parameter of IC, significantly decreased immediately after both R‐EX and S‐EX compared with that before each exercise (both *P*s < 0.05). The degree of changes in IC following exercise did not differ between the two protocols. By contrast, the results of the present study showed that R‐EX may have more beneficial effects on cardiac and perceptual responses than S‐EX. Therefore, the present study determined that R‐EX changes postexercise IC similar to S‐EX. We suggest that R‐EX can be used as safe and effective exercise protocol to improve cognitive function in various populations.

## Graphical abstract

Aerobic exercise improves postexercise cognitive function. Effective aerobic exercise protocols for improving cognitive function are poorly understood. A repeated bout protocol of aerobic exercise can be used as safe and effective exercise protocol to improve cognitive functioin.

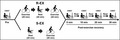

## 1 INTRODUCTION

Cognitive inhibitory control (IC) is defined as the suppression of behavior in response to either an internal or external stimuli (Ozonoff & Strayer, 1997), which is necessary to prevent the implementation of an unrequired action (Coxon, Stinear, & Byblow, 2007). We previously reported that an acute bout of aerobic exercise improved postexercise IC (Hashimoto et al., 2018; Tanaka et al., 2018; Tsukamoto et al., 2016a, 2016b, 2018; Tsukamoto, Takenaka, et al., 2017) and demonstrated that the degree of postexercise IC improvements is associated with an increase in exercise volume (Tsukamoto, Takenaka, et al., 2017), which is the product of exercise intensity and duration.

Many guidelines have recommenced moderate‐intensity physical activity for a minimum of 30 min per day in order to obtain the substantial benefits of exercise in most populations (e.g., Garber et al., 2011). However, longer duration of exercise is a barrier to perform exercise habitually (Trost, Owen, Bauman, Sallis, & Brown, 2002). In this regard, repeated bouts of aerobic exercise (R‐EX) are performed with a rest interval during this protocol, which is beneficial for improving exercise adherence compared to the prolonged protocol, such as a single bout of aerobic exercise (S‐EX), because of shorter durations per exercise bouts (Jakicic, Winters, Lang, & Wing, 1999). In addition to this benefit, R‐EX improves body composition (e.g., reduced body weight and body fat mass), cardiovascular function (e.g., reduced blood pressure), and exercise tolerance (e.g., increased peak oxygen consumption [VO_2 peak_]) similarly to S‐EX in various populations, including older individuals and patients with chronic disease (Jakicic et al., 1999; Murphy & Hardman, 1998; Schmidt, Biwer, & Kalscheuer, 2001). Furthermore, previous laboratory‐based studies reported that aerobic exercise‐induced increases in systemic fatty acid metabolic parameters (e.g., increased blood free fatty acid levels and whole‐body fatty acid oxidation) were greater following R‐EX than following S‐EX (Goto, Ishii, Mizuno, & Takamatsu, 2007; Goto, Tanaka, Ishii, Uchida, & Takamatsu, 2011; Kurobe, Nakao, Nishiwaki, & Matsumoto, 2017; Stich et al., 2000); therefore, these acute responses support the beneficial long‐term effects of R‐EX on many health parameters. Taking these findings into consideration, R‐EX is an effective exercise protocol for improving public health. However, to the best of our knowledge, the effect of R‐EX on cognitive function remains unknown.

Previous studies reported that increased duration of aerobic exercise is useful in enhancing postexercise improvements in cognitive functions (Gejl et al., 2018; Shibasaki et al. 2019), suggesting that aerobic exercise duration is an important variable in determining the degree of exercise‐induced improvements in cognitive functions. However, Chang et al. (2015) reported that the shortening in the Stroop task‐measured response time immediately after moderate‐intensity exercise was greater following a 20‐min exercise duration bout of exercise than following a 10 or 40‐min duration bout of exercise. Furthermore, in a recent study, we reported that, although postexercise IC improvements induced by moderate‐intensity exercise were slightly more sustained following a 40‐min bout than a 20‐min bout of exercise, the degree of the IC improvements throughout experimental session did not differ between the two duration protocols (Tsukamoto, Takenaka, et al., 2017). These previous findings suggest that a 20‐min bout of moderate‐intensity exercise may be sufficient in improving postexercise IC. Given these findings, one possibly can expect that R‐EX with two 20‐min bouts may be more effective for improving postexercise IC compared to S‐EX with a 40‐min bout, resulting from an additive effect induced by the two bouts. To test this possibility, in the present study, we compared the degree of changes in IC following R‐EX and S‐EX.

## 2 METHODS

### 2.1 Participants

Twenty healthy, young men (age: 21.0 ± 0.4 years, body height: 172.6 ± 0.7 cm, body weight: 64.0 ± 2.0 kg, VO_2 peak_: 45.9 ± 1.0 ml min^−1^ kg^−1^) participated in this study. Prior to this study, we calculated the required sample size utilizing an effect size (0.30), an α‐level of 0.05, and a β‐level of 0.2 (80% power), based on the data of our previous study (Tsukamoto et al., 2016a). The calculated necessary number of subjects was 15; therefore, this study has an adequate sample size to ensure statistical power and sensitivity. The subjects were recreationally active and participated in physical exercise (e.g., aerobic exercise) for 2–4 hr per week. The subjects were free of any known neurological, cardiovascular, and pulmonary disorders, as well as free from color‐blindness and abnormal vision. The subjects were instructed to avoid strenuous physical activity in the 24 hr prior to each experiment. Each subject also abstained from food, caffeine, and alcohol for 12 hr prior to each experiment, and was not taking any medications that may affect cognitive performance. All subjects provided written informed consent upon having the experimental procedures and potential risks described. This study was approved by the Ethics Committee of Ritsumeikan University and conducted according to the Declaration of Helsinki.

### 2.2 Experimental conditions

**Figure 1:**
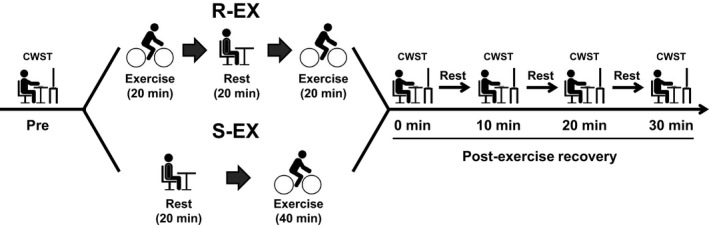
Experimental procedures of repeated bouts (R‐EX) and a single bout (S‐EX) of moderate‐intensity exercise. R‐EX consisted of two 20‐min bouts of moderate‐intensity exercise (i.e., 60% VO_2 peak_) for 20 min_,_ which were separated by a 20‐min sitting rest. S‐EX consisted of a once‐off 40‐min bout of moderate‐intensity exercise. The color‐word Stroop task (CWST) was administered before exercise (i.e., Pre), immediately after exercise, and every 10 min during the 30‐min postexercise recovery period

Experimental procedures of R‐EX and S‐EX protocols are presented in Figure 1. All subjects completed both R‐EX and S‐EX sessions on a cycle ergometer in a randomized and counterbalanced order. The two experiment days were separated by at least 72 hr, and the maximum interval between the conditions was 7 days. R‐EX consisted of two 20‐min bouts of moderate‐intensity exercise (i.e., 60% VO_2 peak_) for 20 min_,_ which were separated by a 20‐min sitting rest. S‐EX consisted of a once‐off 40‐min bout of moderate‐intensity exercise. Mean value of exercise volume for both protocols was 355 ± 9 kJ.

### 2.3 Experimental design

All subjects completed a familiarization visit where they practiced the three types of the color‐word Stroop task (CWST) for each a minimum of 10 times until they achieved consistent scores. During this visit, the subjects also performed a ramp‐incremental test on a cycle ergometer to determine their VO_2 peak_, which was used to calculate the workload of moderate‐intensity exercise for both R‐EX and S‐EX.

On the day of the experiment, the subjects practiced the three CWST types for each a minimum of five times before experimental session to minimize learning effect. Then, the subjects rested for 5 min before undergoing measurements of cardiovascular parameters and blood metabolites. Once this was completed, the subjects performed the CWST before exercise session (i.e., preexercise). In order to ensure consistency in time intervals in both protocols between CWSTs before and after exercise session, S‐EX included a 20‐min rest period in the sitting position following the preexercise CWST before exercise session.

Subjects completed either R‐EX or S‐EX. The CWST was performed again immediately after completion of the exercise session and then was repeated three times at 10‐min intervals during the 30‐min postexercise recovery period to evaluate the sustainable effect of postexercise IC improvements induced by R‐EX and S‐EX, as in our previous studies (Tanaka et al., 2018; Tsukamoto et al., 2016a, 2018).

Cardiovascular (i.e., heart rate [HR] and mean arterial pressure [MAP]) and perceived exertion (i.e., rating of perceived exertion [RPE] and leg discomfort) parameters were measured eight times during both R‐EX and S‐EX. During the R‐EX, cardiovascular and perceived exertion parameters were measured every 5 min (i.e., 5, 10, 15, and 20 min during each exercise bout) over each 20‐min exercise bout. During the S‐EX, cardiovascular and perceived exertion parameters were measured every 5 min throughout a once‐off 40‐min exercise bout (i.e., 5, 10, 15, 20, 25, 30, 35, and 40 min during an exercise bout). Additionally, cardiovascular and blood metabolites parameters were measured immediately before all five CWSTs to assess the effects of cardiovascular and metabolic condition on IC. Felt arousal scale (FAS) and visual analog scale (VAS) were measured immediately after the five CWSTs to assess the effect of psychological conditions on IC.

### 2.4 Ramp‐incremental test

A ramp‐incremental test was performed to determine VO_2_ peak on a cycle ergometer, as previously described (Tanaka et al., 2018; Tsukamoto et al., 2016a, 2016b, 2018; Tsukamoto, Takenaka, et al., 2017). In brief, subjects performed 3 min of baseline cycling at 30 W, after which the workload was increased at a rate of 30 W/min until the limit of tolerance. The subjects were asked to maintain a cadence of 60 rpm. During this test, breath‐by‐breath pulmonary gas‐exchange data were collected and averaged every 10 s (AE‐310S; Minato Medical Science). The VO_2_ peak was determined as the highest 30‐s mean value attained prior to exhaustion.

### 2.5 Cardiovascular parameters

HR was measured continuously via telemetry (RS400; Polar Electro Japan). Systolic blood pressure and diastolic blood pressure were measured using a mercury manometer (FC‐110ST; Focal). MAP was calculated as ([systolic blood pressure−diastolic blood pressure]/3 + diastolic blood pressure).

### 2.6 Blood metabolites

Blood glucose and lactate levels were measured using a glucose (Medisafe FIT Blood Glucose Meter; Terumo) and lactate analyzer (Lactate Pro 2; Arkray), respectively.

### 2.7 Perceived exertion

The Borg's RPE scale was measured to assess perceived exertion expended during exercise, and this scale was ranged from 6 (no exertion) to 20 (maximal exertion) (Borg, 1982). The Borg category‐ratio scale was measured to assess leg discomfort during exercise, and this scale was ranged from 0 (nothing at all) to 10 (absolute maximum) (Borg, 1982).

### 2.8 Psychological conditions

FAS was measured to assess arousal during the CWST, and this scale is a 6‐point, single‐item scale ranging from 1 (low arousal) to 6 (high arousal) (Svebak & Murgatroyd, 1985). VAS was measured to assess psychological conditions during the CWST, and these scales consisted of questions of three psychological types that assess mental fatigue, the ability to concentrate, and motivation. Each VAS was labeled from 0 mm (i.e., not at all) to 100 mm (i.e., extremely). The subjects drew lines to indicate their response.

### 2.9 CWST

The CWST was administered to determine IC, which is a well‐known paradigm for investigating domains of cognitive performance that depend on IC (Stroop, 1935). The stimulus words of the CWST were consisted of four color names (“RED,” “YELLOW,” “GREEN,” and “BLUE”), and they were presented on a 98‐inch display. All words were written in Japanese for Japanese subjects. The subjects were required to press the color‐labeled keypad that corresponded to the text meaning of the stimulus word. The CWST consisted of two color text tasks and one control black text task. The congruent task, which is a facilitated task, displayed the color names presented in the same‐colored text. The neutral task, which is a control task, displayed the color names presented in black text. The incongruent task, which is an interference task, displayed the color names presented in a different‐colored text. One trial of each CWST task consisted of 24 stimulus words, which were presented in a random order. The CWST for each time point consisted of three trials for all three tasks in a random and counterbalanced order. The reaction time and response accuracy for each trial were calculated as the average of the three trials for each CWST task. IC assessed using the reverse‐Stroop interference score, which is the difference between the reaction times for the neutral and incongruent tasks. The reverse‐Stroop interference score is more appropriate for calculating the interference effect when measured by the manual response modality (i.e., manual keypad pressing) than the Stroop interference score (Ikeda, Hirata, Okuzumi, & Kokubun, 2010). The reverse‐Stroop interference score was calculated as ([reaction time of incongruent task−reaction time of neutral task]/reaction time of neutral task × 100) (Ikeda et al., 2010).

### 2.10 Statistical analysis

The data are expressed as the mean ± *SEM*. The mean values of cardiovascular (i.e., HR and MAP) and perceptual (i.e., RPE and leg discomfort) responses during R‐EX and S‐EX were compared using a paired Student's *t* test. Changes in measured variables throughout experimental session between the two conditions were analyzed using two‐way (condition × time) repeated‐measures analysis of variance. If the sphericity assumption was not met, Greenhouse–Geisser corrections were used. Specific differences were identified with a paired Student's *t* test or a Bonferroni post‐hoc test. The statistical significance level was defined at *p* < .05. All statistical analyses were conducted using IBM SPSS software (Ver. 19.0, IBM Corp).

Cohen's *d* effect size using the pooled standard deviation was calculated to determine the magnitude of a difference in the reverse‐Stroop interference score between before (i.e., baseline and preexercise) and after (i.e., immediately postexercise and postexercise recovery periods) R‐EX and S‐EX. The Cohen's *d* effect size were interpreted as small (0.20–0.49), moderate (0.50–0.79), and large (>0.80) (Cohen, 1992). Partial eta squared (*η*_p_^2^) values were determined as a measure of the effect size for main effects of condition and time or interaction effect.

## 3 RESULTS

### 3.1 Measured variables during exercise session

Mean values of HR (149.7 ± 2.1 and 153.2 ± 2.8 bpm) and MAP (95.0 ± 1.4 and 97.2 ± 1.7 mmHg) throughout exercise did not differ significantly between R‐EX and S‐EX. Similarly, mean values of RPE (13.6 ± 0.3 and 13.9 ± 0.3) and lower limb discomfort (4.9 ± 0.3 and 5.0 ± 0.3) throughout exercise did not differ significantly between R‐EX and S‐EX. However, mean RPE during the second bout of R‐EX was significantly lower than that during the latter 20‐min of S‐EX (14.0 ± 0.3 versus 14.8 ± 0.3, respectively; *p* = .041, *d* = 0.53).

### 3.2 Changes in physiological variables throughout experimental session

Changes in physiological variables throughout R‐EX and S‐EX sessions are shown in Table 1. With regard to cardiovascular responses, HR analysis revealed significant main effects for time (*F*_(2.42, 45.95)_ = 1105.21, *p* < .001, *η*_p_^2^ = 0.98) and a significant interaction effect (*F*_(2.35, 44.63)_ = 3.80, *p* = .024, *η*_p_^2^ = 0.18). A trend against significance was also obtained in main effect for condition (*F*_(1, 19)_ = 3.40, *p* = .081, *η*_p_^2^ = 0.15). HR significantly increased immediately after R‐EX and S‐EX compared with that before each exercise (both *P*s < 0.001, *d* = 11.93 and 9.53, respectively), and the increased HR remained significant until the 30‐min postexercise recovery period for both protocols (all *P*s < 0.01, *d* = 1.33 to 3.58). Although HR immediately after exercise did not differ significantly between R‐EX and S‐EX (158.2 ± 2.2 and 159.6 ± 2.9 bpm, respectively), HR at the 10‐min postexercise recovery period was significantly lower for R‐EX than for S‐EX (82.5 ± 1.8 versus 88.5 ± 1.7 bpm, respectively; *p* = .005, *d* = 0.79). MAP analysis revealed a significant main effect for time (*F*_(1.97, 37.39)_ = 53.89, *p* < .001, *η*_p_^2^ = 0.74). MAP significantly increased immediately after R‐EX and S‐EX compared with that before each exercise (both *P*s < 0.001, *d* = 1.46 and 1.31, respectively).

**Table 1:** Changes in cardiovascular and blood metabolite responses throughout experimental sessions in repeated bouts (R‐EX) and a single bout (S‐EX) of moderate‐intensity exercise

	Time points					*p* values		
	Pre‐EX	Post‐EX 0 min	Post‐EX 10	Post‐EX 20	Post‐EX 30	Condition	Time	Interaction
Heart rate (bpm)								
R‐EX	66.5 ± 1.0	158.2 ± 2.2^a^	82.5 ± 1.8^a^, ^b^, *	78.1 ± 1.6^a^, ^b^, ^c^	74.8 ± 1.7^a^, ^b^, ^c^	.081	**<.001**	**.024**
S‐EX	66.8 ± 1.0	159.6 ± 2.9^a^	88.5 ± 1.7^a^, ^b^	80.6 ± 1.4^a^, ^b^, ^c^	76.4 ± 1.1^a^, ^b^, ^c^			
MAP (mmHg)								
R‐EX	86.1 ± 1.4	96.0 ± 1.6^a^	85.6 ± 1.3^b^	84.7 ± 1.1^b^	84.7 ± 1.2^b^	.350	**<.001**	.980
S‐EX	85.7 ± 1.4	95.8 ± 2.0^a^	85.2 ± 1.4^b^	84.3 ± 1.3^b^	83.8 ± 1.2^b^			
Blood glucose (mg/dl)								
R‐EX	97.7 ± 1.7	84.4 ± 2.1^a^	91.3 ± 1.8^a^, ^b^	90.4 ± 1.7^a^	91.6 ± 1.3^a^, ^b^	.794	**<.001**	.147
S‐EX	96.5 ± 1.9	88.0 ± 2.4	92.3 ± 2.3	91.1 ± 1.9^a^	89.7 ± 1.8^a^			
Blood lactate (mM)								
R‐EX	1.0 ± 0.0	2.8 ± 0.3^a^	1.7 ± 0.2^a^, ^b^	1.4 ± 0.1^b^, ^c^	1.3 ± 0.1^b^, ^c^	.517	**<.001**	**.033**
S‐EX	1.0 ± 0.0	3.2 ± 0.3^a^	1.7 ± 0.1^a^, ^b^	1.4 ± 0.1^b^, ^c^	1.2 ± 0.1^b^, ^c^			

Note

Values are presented as Mean ± *SEM*. Bold *P* values indicate a significant main effect of time and a significant interaction effect (*p* < .05 for all).

Abbreviations: MAP; mean arterial pressure, Pre‐EX; before exercise, Post‐EX 0; immediately after exercise, Post‐EX 10; 10‐min postexercise recovery period, Post‐EX 20; 20‐min postexercise recovery period, Post‐EX 30; 30‐min postexercise recovery period.

^a^ Significant difference (*p* < .05) from Pre‐EX.

^b^ Significant difference (*p* < .05) from Post‐EX 0.

^c^ Significant difference (*p* < .05) from Post‐EX 10.

* Significant difference (*p* < .01) between R‐EX and S‐EX.

With regard to blood metabolite responses, blood glucose analysis revealed a significant main effect for time (*F*_(1.87, 35.53)_ = 17.92, *p* < .001, *η*_p_^2^ = 0.49). Blood glucose significantly decreased immediately after R‐EX compared with that before exercise (*p* = .015, *d* = 1.53), the decreased blood glucose remained significant until the 30‐min postexercise recovery period for this protocol (all *P*s < 0.05, *d* = 0.80–0.95). Blood glucose immediately after S‐EX was a lower trend than that before exercise (*p* = .066, *d* = 0.31), but did not reach statistical significance. Blood glucose levels at 20 and 30 min after S‐EX were significantly lower than that before exercise (both *P*s < 0.05, *d* = 0.63 and 0.82, respectively). Blood lactate analysis revealed a significant main effect for time (*F*_(1.21, 23.03)_ = 51.85, *p* < .001, *η*_p_^2^ = 0.73) and a significant interaction effect (*F*_(1.73, 32.91)_ = 4.02, *p* = .033, *η*_p_^2^ = 0.17). Blood lactate significantly increased immediately after R‐EX and S‐EX compared with that before each exercise (both *P*s < 0.001, *d* = 2.18 and 2.21, respectively), and the increased blood lactate remained significant until the 10‐min postexercise recovery period for both protocols (both *P*s < 0.01, *d* = 1.33 and 1.49, respectively). Blood lactate immediately after exercise was a lower trend for R‐EX than for S‐EX (*p* = .066, *d* = 0.31), but did not reach statistical significance.

### 3.3 Changes in IC throughout experimental session

Changes in reaction times and response accuracies on three types of the CWST throughout R‐EX and S‐EX sessions are summarized in Table 2. Reaction time analyses for each CWST type revealed a significant main effect for time (*F*_(4, 76)_ = 2.85, *p* = .030, *η_p_* ^2^ = 0.13 for congruent task; *F*_(4, 76)_ = 11.80, *p* < .001, *η*_p_^2^ = 0.38 for neutral task; *F*_(2.75, 52.25)_ = 28.04, *p* < .001, *η*_p_^2^ = 0.60 for incongruent task). Congruent reaction time significantly shortened immediately after only an R‐EX compared with that before exercise (*p* = .007, *d* = 0.29). Neutral and incongruent reaction times significantly shortened immediately after R‐EX and S‐EX compared with those before each exercise (all *P*s < 0.05, *d* = 0.26–0.61), the decreased neutral and incongruent reaction times remained until the 10‐min postexercise recovery period (all *P*s < 0.05, *d* = 0.26–0.50). Response accuracy analyses for all three tasks revealed no significant main effects for time and condition or no significant interaction effects.

**Table 2:** Summary of reaction times and response accuracies of three types of the color‐word Stroop task (CWST) throughout R‐EX and S‐EX sessions

	Reaction time (msec)		Response accuracy (%)	
	R‐EX	S‐EX	R‐EX	S‐EX
Congruent task				
Pre‐EX	9,462 ± 394	9,206 ± 432	95.2 ± 0.9	96.6 ± 0.6
Post‐EX 0	8,967 ± 363	8,961 ± 375	96.1 ± 0.9	96.5 ± 0.6
Post‐EX 10	8,880 ± 367^a^	8,999 ± 424	96.4 ± 0.8	97.1 ± 0.5
Post‐EX 20	9,260 ± 370	9,027 ± 376	95.7 ± 0.9	96.5 ± 0.7
Post‐EX 30	9,324 ± 406	9,133 ± 403	96.5 ± 0.6	96.1 ± 0.8
Neutral task				
Pre‐EX	9,959 ± 430	9,972 ± 433	97.0 ± 0.6	97.1 ± 0.6
Post‐EX 0	9,285 ± 396^a^	9,209 ± 424^a^	97.8 ± 0.4	97.5 ± 0.6
Post‐EX 10	9,485 ± 399^a^	9,335 ± 393^a^	96.9 ± 0.5	97.7 ± 0.7
Post‐EX 20	9,798 ± 431	9,541 ± 403	97.6 ± 0.6	96.8 ± 0.5
Post‐EX 30	9,818 ± 401	9,546 ± 413	96.4 ± 0.8	97.9 ± 0.5
Incongruent task				
Pre‐EX	10,970 ± 465	10,946 ± 459	96.6 ± 0.5	96.6 ± 0.5
Post‐EX 0	9,917 ± 444^a^	9,805 ± 414^a^	96.2 ± 0.8	97.1 ± 0.8
Post‐EX 10	10,296 ± 414^a^	10,023 ± 415^a^	96.2 ± 0.7	97.2 ± 0.5
Post‐EX 20	11,697 ± 451^b^, ^c^	10,354 ± 442^b^	96.7 ± 0.7	96.0 ± 0.9
Post‐EX 30	11,755 ± 431^b^	10,417 ± 433^a^, ^b^	95.9 ± 0.7	96.9 ± 0.7

Note

Values are presented as means ± *SEM*. Analyses for reaction times of three CWST types revealed a significant main effect for time (*p* < .01 for all).

^a^ Significant difference (*p* < .05) from Pre‐EX.

^b^ Significant difference (*p* < .05) from Post‐EX 0.

^c^ Significant difference (*p* < .05) from Post‐EX 10.

**Figure 2:**
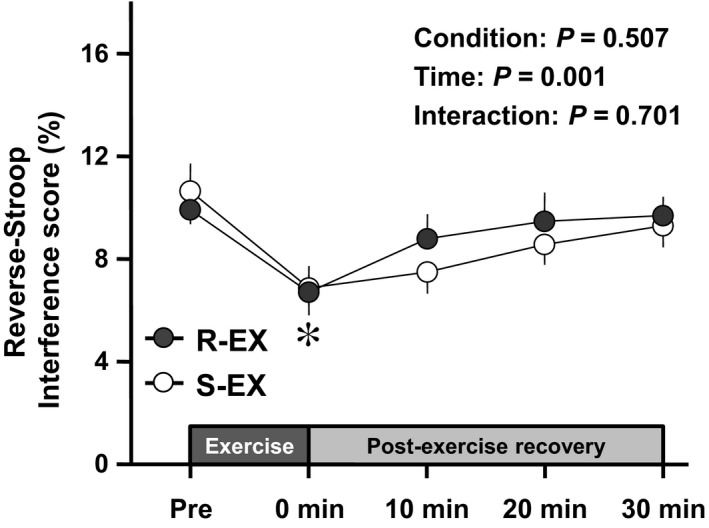
Changes in the reverse‐Stroop interference score throughout R‐EX and S‐EX sessions. Values are presented as Mean ± *SEM*. ^*^Significant difference (*p* < .05) from Pre of both R‐EX and S‐EX

Changes in the reverse‐Stroop interference score (i.e., IC) throughout R‐EX and S‐EX sessions are presented in Figure 2. The reverse‐Stroop interference score analysis revealed a significant main effect for time (*F*_(4, 76)_ = 5.10, *p* = .001, *η*_p_^2^ = 0.21). The reverse‐Stroop interference score significantly decreased immediately after R‐EX and S‐EX compared with that before each exercise (both *P*s < 0.05, *d* = 0.96 and 0.87, respectively).

### 3.4 Changes in psychological conditions throughout experimental session

Changes in psychological conditions throughout R‐EX and S‐EX sessions are shown in Table 3. FAS‐measured arousal analysis revealed significant main effects for condition (*F*_(1, 19)_ = 6.78, *p* = .017, *η*_p_^2^ = 0.26) and time (*F*_(2.16, 40.94)_ = 17.51, *p* < .001, *η*_p_^2^ = 0.48) and a significant interaction effect (*F*_(2.09, 39.62)_ = 3.44, *p* = .040, *η*_p_^2^ = 0.15). Arousal significantly increased immediately after R‐EX and S‐EX compared with that before each exercise (both *P*s < 0.01, *d* = 1.00 and 1.70, respectively), and the increased arousal remained until the 10‐min postexercise recovery period in only an S‐EX (*p* = .002, *d* = 0.99). Arousal levels immediately and 10 min after exercise were significantly lower for R‐EX than for S‐EX (both *P*s < 0.01, *d* = 0.56 and 0.49, respectively). All three VAS‐measured psychological conditions analyses revealed significant effects for time (*F*_(4, 76)_ = 27.47, *p* < .001, *η*_p_^2^ = 0.59 for mental fatigue; *F*_(4,68)_ = 7.11, *p* < .001, *η*_p_^2^ = 0.27 for concentration; *F*_(4 ,76)_ = 3.75, *p* = .008, *η*_p_^2^ = 0.17 for motivation). Mental fatigue significantly increased immediately after R‐EX and S‐EX compared with that before exercise (both *P*s < 0.001,* d* = 2.48 and 2.71, respectively), and the increased mental fatigue remained until the 30‐min postexercise recovery period for both protocols (all *P*s < 0.05, *d* = 0.93–1.45). There were no significant differences for concentration and motivation between conditions or time points throughout experimental session.

**Table 3:** Changes in psychological conditions for the CWST throughout R‐EX and S‐EX sessions

	Time points					*p* values		
	Pre‐EX	Post‐EX 0	Post‐EX 10	Post‐EX 20	Post‐EX 30	Condition	Time	Interaction
Arousal								
R‐EX	1.8 ± 0.2	3.0 ± 0.3^a^, ^*^	2.3 ± 0.2*	2.3 ± 0.2	2.3 ± 0.3	**.017**	**<.001**	**.040**
S‐EX	1.9 ± 0.2	3.7 ± 0.3^a^	2.9 ± 0.3^a^, ^b^	2.4 ± 0.3^b^, ^c^	2.5 ± 0.2^a^, ^b^			
Mental fatigue (mm)								
R‐EX	20.7 ± 3.3	64.7 ± 4.6^a^	49.1 ± 5.5^a^	49.1 ± 6.4^a^	46.1 ± 6.3^a^, ^b^	.696	**<.001**	.619
S‐EX	24.3 ± 3.9	70.4 ± 3.8^a^	50.0 ± 4.1^a^, ^b^	46.1 ± 6.0^a^, ^b^	45.6 ± 6.1^a^, ^b^			
Concentration (mm)								
R‐EX	56.3 ± 4.5	69.3 ± 4.0	60.7 ± 4.3	53.9 ± 4.0^b^	54.1 ± 3.4^b^	.133	**<.001**	.365
S‐EX	61.6 ± 5.0	78.0 ± 3.6	59.8 ± 3.3^b^	52.7 ± 4.7^b^	58.6 ± 4.2^b^			
Motivation (mm)								
R‐EX	65.2 ± 4.3	71.6 ± 3.4	65.9 ± 4.1	64.5 ± 4.3	66.1 ± 3.6	.937	**.008**	.381
S‐EX	67.3 ± 3.9	74.6 ± 4.4	67.5 ± 4.2	61.2 ± 5.3	63.3 ± 4.7			

Note

Values are presented as Mean ± *SEM*. Bold *P* values indicate significant main effects of time and/or condition and a significant interaction effect (*p* < .05 for all).

^a^ Significant difference (*p* < .05) from Pre‐EX.

^b^ Significant difference (*p* < .05) from Post‐EX 0.

^c^ Significant difference (*p* < .05) from Post‐EX 10.

* Significant difference (*p* < .01) between R‐EX and S‐EX.

## 4 DISCUSSION

In this study, we expected that R‐EX would be more effective in changing postexercise IC than S‐EX because of an additive effect induced by the two bouts of exercise. Unexpectedly, the primary finding of the present study was that although the IC (i.e., the reverse‐Stroop interference score) significantly changed following R‐EX and S‐EX, the degree of changes in IC throughout the experimental session did not differ between the two protocols. In a potential explanation of this finding, R‐EX was separated by a 20‐min rest interval, and thus, the rest interval caused a wash‐out of the IC changes induced by the first bout of exercise. Our previous study determined that although postexercise IC improvements induced by moderate‐intensity exercise were slightly more sustained following a 40‐min bout than a 20‐min bout of exercise, the degree of the exercise‐induced IC improvements throughout the 30‐min postexercise recovery period did not differ between the two duration protocols (Tsukamoto, Takenaka, et al., 2017). Therefore, the changes in IC following R‐EX may have been induced only by the effect of the second bout due to the wash‐out of the effect of the first bout. These findings suggest that although R‐EX changes postexercise IC similar to S‐EX, it may have not the additive effect that changes postexercise IC.

Our previous studies demonstrated that the level of aerobic exercise‐induced increase in blood lactate is associated with the degree of postexercise IC improvements (Hashimoto et al., 2018; Tsukamoto et al., 2016b), potentially caused by increasing cerebral lactate metabolism (Hashimoto et al., 2018). The result of the present study showed a significant interaction effect for blood lactate with a trend against significance for a lower blood lactate immediately after R‐EX than that immediately after S‐EX. Nevertheless, the degree of changes in blood lactate throughout experimental session did not differ between R‐EX and S‐EX. Therefore, the results of the blood lactate may help in understanding the present findings that the degree of postexercise IC changes was similar between R‐EX and S‐EX.

Changes in perceptual responses such as arousal are associated with increases in cerebral neural activity and cognitive function (Byun et al., 2014). Additionally, our previous study determined that high‐intensity interval exercise was more effective in improving IC than moderate‐intensity continuous exercise, which was correspond to a higher arousal response for high‐intensity interval exercise than that for moderate‐intensity continuous exercise. The result of the present study showed that although FAS‐measured arousal increased following R‐EX and S‐EX, the increase was greater following S‐EX than following R‐EX. Therefore, arousal may be insufficient to explain the similar changes in IC following R‐EX and S‐EX with a same intensity (i.e., moderate intensity).

An elevation in perceived exertion response during exercise can be considered a barrier to exercise participation among some individuals (Trost et al., 2002); in other words, a reduction in perceived exertion response during exercise may contribute to improve exercise adherence. Kurobe et al. (2017) reported that RPE at the end of exercise was a lower trend for R‐EX than for S‐EX (11.0 ± 0.6 versus 11.4 ± 0.7, respectively). In the present study, although the mean value of RPE throughout exercise did not differ between R‐EX and S‐EX, the mean value of RPE during the second bout of R‐EX was significantly lower than that during the latter 20 min of S‐EX. These findings suggest that R‐EX may be a beneficial strategy for performing exercise habitually, potentially by mitigating the perceived exertion response during exercise, which may contribute to improved exercise adherence.

Previous studies determined that HR recovery after exercise was greater following R‐EX than following S‐EX (Cunha, Midgley, Pescatello, Soares, & Farinatti, 2016; Jones, Taylor, Lewis, George, & Atkinson, 2009). In the present study, although HR immediately after exercise did not differ significantly between R‐EX and S‐EX, HR at 10 min after exercise was significantly lower for R‐EX than for S‐EX. Moreover, a reduction in the HR between immediately and 10 min after exercise was greater for R‐EX than for S‐EX (Δ75.7 ± 1.7 versus 71.1 ± 2.1 bpm, *p* = .010). The faster HR recovery after R‐EX than that of S‐EX may be beneficial in reducing the likelihood of a cardiovascular event following exercise in older individuals and patients with chronic diseases (Cole, Blackstone, Pashkow, Snader, & Lauer, 1999; Jouven et al., 2005). Additionally, previous studies determined that aerobic exercise‐induced reduction of arterial stiffness was sustained much better following R‐EX than following S‐EX (Wang, Zhang, Zhu, Wu, & Yan, 2014; Zheng et al., 2015). Therefore, postexercise cardiovascular responses following R‐EX may be safer and more advantageous than those following S‐EX.

The present study has several limitations. First, although our previous study reported that control sitting rest (e.g., 45 min) has no effect on changes in IC (Tsukamoto, Suga, et al., 2017; Tsukamoto, Takenaka, et al., 2017), we did not include the control condition (i.e., a 60‐min sitting rest) in the experimental protocols of the present study. Thus, we could not completely exclude some effects (e.g., learning effect) from postexercise IC changes induced by R‐EX and S‐EX; thus, this study was focused only on the comparison of the degree of postexercise IC changes between the two protocols. Second, although we employed the two 20 min bouts with a 20‐min rest interval for R‐EX, the R‐EX has been examined in laboratory‐based previous studies with various protocols using longer or shorter exercise bouts (Cunha et al., 2016; Goto et al., 2007; Stich et al., 2000; Wang et al., 2014; Zheng et al., 2015) and/or rest interval (Cunha et al., 2016; Goto et al., 2007; Stich et al., 2000). Given the results of the present study, we hypothesized that a 20‐min rest interval during R‐EX might result in wash‐out of postexercise IC changes following the first bout; therefore, a rest interval shorter than 20 min may mitigate the wash‐out effect. Additionally, several previous studies employed three 10‐min bouts (which are separated by two rest intervals for 10 min) for the R‐EX protocol (Goto et al., 2011; Jones et al., 2009; Kurobe et al., 2017). Further studies are needed to determine the effective protocols of R‐EX on postexercise IC changes. Third, although we recruited healthy young males, safe and effective exercise programs to improve cognitive function are generally more important in older individuals and patients with chronic diseases than in healthy young populations. Further studies are needed to determine the effect of R‐EX on postexercise IC changes in various populations.

In a perspective, Goto et al. (2007) found that an exercise‐induced increase of circulating 3‐hydroxybutyrate was greater following R‐EX than following S‐EX. The 3‐hydroxybutyrate may be an important regulator for improvement in cognitive function induced by long‐term intervention (Newman et al., 2017), potentially via mediating the expression of brain health‐related markers, such as brain‐derived neurotrophic factor (Hu et al., 2018; Sleiman et al., 2016). Although the long‐term effects of R‐EX on cognitive function and brain health remain unknown, these previous findings suggest that R‐EX may have the underlying potential to improve cognitive function and brain health more than S‐EX. To elucidate this, further studies are needed to examine the effects of long‐term R‐EX on cognitive function and brain health parameters.

## 5 CONCLUSIONS

This study is the first to determine the potential impact of R‐EX on cognitive function. The present study determined that the degree of changes in IC following exercise was similar between R‐EX and S‐EX. In addition, the findings of the present study and those of previous studies suggest that R‐EX may have more beneficial effects on certain factors, such as cardiovascular and perceptual responses, than S‐EX. Therefore, we conclude that R‐EX can be used as a safe and effective exercise protocol to improve cognitive function, in addition to physical function in various populations.

## CONFLICT OF INTEREST

The authors declare no conflict of interest.

## AUTHOR CONTRIBUTIONS

Take S. and Tada S. conceived and designed the experiment, analyzed the data, and wrote the manuscript. Take S., Tada S., H.T., K.T., and K.D. performed experiments. Take.S., Tada.S., H.T., K.T., K.D., T.H., and T.I. interpreted results of experiments. T.H. and T.I. edited and revised the manuscript. All authors have read and approved the manuscript.
